# *EPAS1* gene variants are associated with sprint/power athletic performance in two cohorts of European athletes

**DOI:** 10.1186/1471-2164-15-382

**Published:** 2014-05-18

**Authors:** Sarah Voisin, Pawel Cieszczyk, Vladimir P Pushkarev, Dmitry A Dyatlov, Boris F Vashlyayev, Vladimir A Shumaylov, Agnieszka Maciejewska-Karlowska, Marek Sawczuk, Lidia Skuza, Zbigniew Jastrzebski, David J Bishop, Nir Eynon

**Affiliations:** Institute of Sport, Exercise and Active Living (ISEAL), Victoria University, Melbourne, Australia; Department of Tourism and Recreation, Academy of Physical Education and Sport, Gdansk, Poland; Ural State University of Physical Culture, Chelyabinsk, Russia; Faculty of Physical Culture and Health Promotion, University of Szczecin, Szczecin, Poland; Cell Biology Department, Faculty of Biology, University of Szczecin, Szczecin, Poland; Murdoch Childrens Research Institute, The Royal Children’s Hospital, Melbourne, Australia

**Keywords:** Athletic performance, EPAS1, Genomics, Genes, Endurance, Sprint

## Abstract

**Background:**

The endothelial PAS domain protein 1 (EPAS1) activates genes that are involved in erythropoiesis and angiogenesis, thus favoring a better delivery of oxygen to the tissues and is a plausible candidate to influence athletic performance. Using innovative statistical methods we compared genotype distributions and interactions of *EPAS1* SNPs rs1867785, rs11689011, rs895436, rs4035887 and rs1867782 between sprint/power athletes (n = 338), endurance athletes (n = 254), and controls (603) in Polish and Russian samples. We also examined the association between these SNPs and the athletes’ competition level (‘elite’ and ‘sub-elite’ level). Genotyping was performed by either Real-Time PCR or by Single-Base Extension (SBE) method.

**Results:**

In the pooled cohort of Polish and Russian athletes, 1) rs1867785 was associated with sprint/power athletic status; the AA genotype in rs1867785 was underrepresented in the sprint/power athletes, 2) rs11689011 was also associated with sprint/power athletic status; the TT genotype in rs11689011 was underrepresented sprint/power athletes, and 3) the interaction between rs1867785, rs11689011, and rs4035887 was associated with sprint/power athletic performance; the combinations of the AA genotype in rs4035887 with either the AG or GG genotypes in rs1867785, or with the CT or CC genotypes in rs11689011, were underrepresented in two cohorts of sprint/power athletes.

**Conclusions:**

Based on the unique statistical model rs1867785/rs11689011 are strong predictors of sprint/power athletic status, and the interaction between rs1867785, rs11689011, and rs4035887 might contribute to success in sprint/power athletic performance.

**Electronic supplementary material:**

The online version of this article (doi:10.1186/1471-2164-15-382) contains supplementary material, which is available to authorized users.

## Background

Maximal oxygen uptake (VO_2max_) refers to the highest rate at which oxygen can be consumed by the body during intense exercise [1], and is, among other factors, an important predictor of elite endurance performance [2]. Studies have shown that the changes in VO_2max_ following exercise training vary markedly between individuals and that ~ 50% of the variance can be explained by genetic factors [3]. Over the last two decades, many Single Nucleotide Polymorphisms (SNPs) have been suggested to influence elite performance and the variability in VO_2max_ increase following exercise training [4]. While most of these SNPs were discovered using the candidate gene approach [5], a more comprehensive, genome-wide linkage approach has identified a genomic region on chromosome 2 that is associated with the VO_2max_ training response. The endothelial PAS domain protein 1 (*EPAS1*) gene was one of the four genes in this region responsible for this linkage [6].

EPAS1 is a transcription factor playing a key role in the Hypoxia Inducible Factor (HIF) pathway in blood, which is responsible for activating gene expression in response to hypoxia [7]. In normal oxygen conditions, EPAS1 is quickly degraded in the cytoplasm. However, when oxygen levels drop, EPAS1 becomes stabilized, translocates to the nucleus and activates genes that are involved in erythropoiesis (e.g. erythropoietin), and angiogenesis (e.g., vascular endothelial growth factor), thus favoring a better delivery of oxygen to the tissues [8]. Delivery of oxygen to skeletal muscles during endurance exercise is viewed a factor limiting VO_2max_[1]. Therefore, as a hypoxia detector and as an activator of improved oxygen delivery to the active tissues, *EPAS1* is a plausible candidate to influence endurance performance.

*EPAS1* SNPs have been previously associated with blood parameters, such as alterations in erythropoietin, hemoglobin and hematocrit [9–11], that are important for success in athletic performance. For instance, Tibetans with the TT genotype in registered SNP (rs) 11689011 had lower hemoglobin concentration compared with their TC counterparts [9]. However, to date, only one study has examined a possible link between SNPs within *EPAS1* and elite athletic performance [12]. This study looked at SNPs and haplotypes within *EPAS1* in elite Australian athletes, stratified to two groups participating in middle-distance (from 50 s to 10 min, n = 242), and long-distance (from ~2 to 10 h, n = 151) events. These groups were compared to a non-athletic control group. The T allele in rs11689011 and the G allele in rs1867785, two SNPs located in the first intron of *EPAS1*, were overrepresented in the group of endurance athletes compared with controls [12]. Furthermore, in the same study, two haplotypes involving rs1867785, rs11689011, rs895436 and rs4035887 were associated with elite endurance performance. While haplotype G (A-T-G-G) was overrepresented in elite endurance athletes, haplotype F (G-C-C-G) was underrepresented in elite endurance athletes compared to controls [12]. Despite these positive findings, and the strong biological rational behind investigating *EPAS1* in relation to elite endurance performance, this is the only genetic association study showing that *EPAS1* SNPs impact performance, and the sprinters consisting primarily of 100–400 m track runners and sprint cyclists were excluded from the analysis. Replication studies are therefore needed to confirm this association, particularly in different populations, and with a larger sample size [13].

Therefore, the aim of this study was to compare genotype distributions and interactions of the *EPAS1* SNPs rs1867785, rs11689011, rs895436, rs4035887 and rs1867782 between sprint/power athletes, endurance athletes, and controls in Polish and Russian cohorts. We also examined the association between the *EPAS1* SNPs and athletic status according to the athletes’ level of competition (‘elite’ and ‘sub-elite’ level). In light of the relationship previously observed between endurance-related phenotypes and SNPs in the *EPAS1*, we hypothesized that *EPAS1* SNPs would be associated with elite endurance performance compared to controls and sprint/power athletes. We did not have specific directional hypotheses for rs895436, rs4035887 and rs1867782 as these SNPs have not previously been associated with any performance and/or endurance-related phenotypes. We did not have any directional hypothesis for rs11689011 either, due to the conflicting results reported for this SNP (the T allele in rs11689011 was associated with endurance athletic status in elite Australians on one hand, and with lower hemoglobin concentrations in Tibetans, on the other hand) [9, 12]. However, in line with the findings of Henderson et al. [12] we did expect the rs1867785 G allele to be associated with elite endurance performance. Finally, we did not have any directional hypotheses for any of the SNPs in relation to sprint/power performance.

## Methods

The study was approved by the Pomeranian Medical University Ethics Committee, Poland, and the Ural State University of Physical Culture, Russia, and written informed consent was obtained from each participant. The study complied with the guidelines set out in the Declaration of Helsinki and the ethics policy of the Szczecin University [14].

### Participants

The athletes and controls were all European Caucasians. The athletes were categorized as either endurance athletes or sprint/power athletes as determined by the distance, duration and energy requirements of their event/sport. All athletes were ranked in the top 10 nationally in their sport discipline and grouped as being either ‘elite-level’ or ‘sub-elite’ based on their best personal performance. Those in the elite group had participated in international competitions such as World and European Championships, and/or Olympic Games, whereas those in the sub-elite group had participated in national competitions only. Details on the number of participants in the elite and sub-elite group are presented in Table 1.

**Table 1:** **Athletes’ description**

	Polish athletes (n = 196)		Russian athletes (n = 394)	
	Elite (n = 122)	Sub-elite (n = 74)	Elite (n = 131)	Sub-elite (n = 263)
ENDURANCE				
Rowing	33	8	7	7
Swimming 800 /1500 m	1	9	1	2
Cycling	11	3	0	0
Skating 3000/5000/10000 m	0	0	9	28
Cross-country skiing	2	0	2	62
Canoeing	9	1	0	0
Walking	0	0	5	9
Triathlon	2	3	0	0
Pentathlon	0	0	0	3
Decathlon	0	0	0	10
Marathon	0	6	0	0
Running 1500/3000/5000 m	7	11	1	2
Total	64	40	25	123
SPRINT/POWER				
Skating 500/1000 m	1	0	6	17
Weightlifting	22	20	44	43
Long jump	5	3	1	0
Sprint 100/200/400 m	25	9	1	4
Swimming 50/100 m	2	0	5	12
Shooting	1	0	0	0
Pole vault	1	2	0	3
Javelin throw	1	0	0	0
Ice hockey	0	0	27	16
Taekwondo	0	0	3	5
Karate	0	0	5	3
Boxing	0	0	9	27
Wrestling	0	0	3	8
Ski cross freestyle	0	0	2	0
Snowboarding	0	0	0	1
Discus throw	0	0	0	1
Total	58	34	106	140

### Polish sample

The sample comprised 198 Polish athletes (all men; mean age ± SD, 28 ± 4.4 y), including 92 elite and sub-elite sprint/power athletes and 106 elite and sub-elite endurance athletes, as well as 428 healthy, unrelated, sedentary controls (all male students of the University of Szczecin; mean age ± SD, 20.8 ± 1.2 y).

### Russian sample

The Russian sample comprised 394 athletes (287 men and 107 women; mean age ± SD, 27.8 ± 9.7 y), including 246 elite and sub-elite sprint/power athletes and 148 elite and sub-elite endurance athletes, as well as 175 healthy unrelated sedentary controls (104 men and 44 women, all students or employees of the Ural State University of Physical Culture; mean age ± SD, 30.2 ± 10.7 y). The description of the Polish and the Russian athletes according to their event/sport is summarized in Table 1.

### Genotyping

#### Polish sample

Genomic DNA was isolated from buccal epithelium using GenElute Mammalian Genomic DNA Miniprep Kit (Sigma, Hamburg, Germany) according to the manufacturer’s instructions. All genetic analyses were performed at the Molecular Biology and Biotechnology Center, Faculty of Biology, University of Szczecin. All samples were genotyped in duplicate using allelic discrimination assays with Taqman® probes (Applied Biosystems, Carlsbad, California, USA) on a CFX96 Touch™ Real-Time PCR Detection System (Bio-Rad, Hercules, California, USA). To discriminate *EPAS1* rs1867782, rs1867785, rs11689011, rs895436 and rs4035887 alleles, TaqMan® Pre-Designed SNP Genotyping Assays were used (assay IDs: C__11639978_1_, C__11639984_10, C___2148918_10, C___2148915_10, C___2162989_10, respectively), including appropriate primers and fluorescently labeled (FAM and VIC) MGB™ probes to detect the alleles. Genotypes were assigned using all of the data from the study simultaneously.

#### Russian sample

Genomic DNA was isolated from buccal epithelium or peripheral blood, during the years 2011–2013, using the Diatom™ DNA Prep kit (Cat. # D 1025, IsoGene Lab Ltd, Russia). Genotyping of five selected SNPs was performed by Single-Base Extension (SBE) method. The sequence surrounding each SNP was obtained from the Genome Reference Consortium Human genome build 37 assembly from the Ensembl Project [15]. The Primer3web software v. 4.0.0 [16] was used for designing the PCR primers. PCR product range was 109–173 bp. SBE primers to detect rs895436, rs11689011, rs1867782, and rs1867785 were designed to anneal on the positive strand immediately adjacent to the single nucleotide variation sites. SBE primer for detection of rs4035887 was designed to anneal on the negative strand. To avoid any non-specific amplification and extension products, all primers were BLASTed against human genome reference sequence. Sets of preselected PCR primer pairs and SBE primers were screened for potential cross-reactivity by using AutoDimer software.

Multiplex PCR was performed in a volume of 15 μL containing 1 × PCR buffer, 1.0 mM MgCl_2_, 0.2 mM dNTPs, 0.7 μM of each primer (5 pairs), 1 unit SmarTaq DNA polymerase (Dialat Ltd, Russia) and 5 ng of template DNA. Thermal cycler conditions were: 95°C for 30 s, 30 cycles of 95°C for 45 s, 60°C for 45 s, 72°C for 60 sec and finally 10 min at 72°C in GeneAmp® PCR System 9700 (Applied Biosystems). Multiplex PCR products were checked for quality and yield by running 3 μl in 2% agarose-TBE gels. 5 μL of PCR products were cleaned with 1 unit of FastAP Thermosensitive Alkaline Phosphatase (TAP) and 10 units of Exonuclease I (both enzymes from Fermentas). Multiplex SBE reaction was performed by using SNaPshot® Multiplex Kit (Applied Biosystems) in 5 μL final volume, including 2.5 μL of SnaPshot Multiplex Ready Reaction Mix, 1.0 μl pooled SBE primers and 1.5 μl of cleaned PCR product (the PCR sequences, the SBE primers and their final concentration can be received from the authors by request). The cycling conditions were 96°C 10 s, 50°C 5 s and 60°C 30 s, during 25 cycles in GeneAmp® PCR System 9700 (Applied Biosystems). To remove the unincorporated ddNTPs, the final product was incubated with 1 unit of TAP (Fermentas). SnaPshot products with GeneScan™ - 120 LIZ™ Size Standard (AB) were diluted in Hi-Di™ Formamide (AB), denatured and separated using an ABI PRISM 310 Genetic Analyzer (AB) with a 47 cm length capillary and POP-4™ polymer (AB). The SnaPshot® Primer Focus® Kit (AB) was used to analyze individual SBE primers for their approximate sizing locations prior to performing the multiplex SBE reaction. Final data were analyzed using the GeneMapper® Software v. 4.1 from Applied Biosystems following the software manual.

K562 DNA High Molecular Weight from Promega Corp. (USA) served as positive control sample. Genetic profile of K562 DNA was following: rs895436 – G/G, rs11689011 – C/C, rs4035887 – T/T (for the negative strand), rs1867785 – G/G, rs1867782 – C/C.

Each of the five PCR products (for the five different SNPs) was formed from five different individuals, and was sequenced in separate reaction using BigDye® Terminator v3.1 Cycle Sequencing Kit (Applied Biosystems) with full coincidence of expected and observed sequences. Sequencing was performed in a second laboratory (Gordiz Ltd. Laboratory, Moscow, Russia), according to latest recommendations [17].

### Genotyping reliability across two laboratories

Genotyping was performed in duplicate in the same Laboratory for accuracy. Two independent investigators have called the genotyping score in each laboratory-100% of the genotypes could be called. For the purpose of results reliability across two laboratories in two different countries (Russia and Poland), different DNA samples (one for each SNP, positive or negative controls) were shipped from Russia to Poland and were genotyped by TaqMan assays. The results of the genotyping were in 100% agreement across the two laboratories.

### Statistical analysis

**Figure 1:**
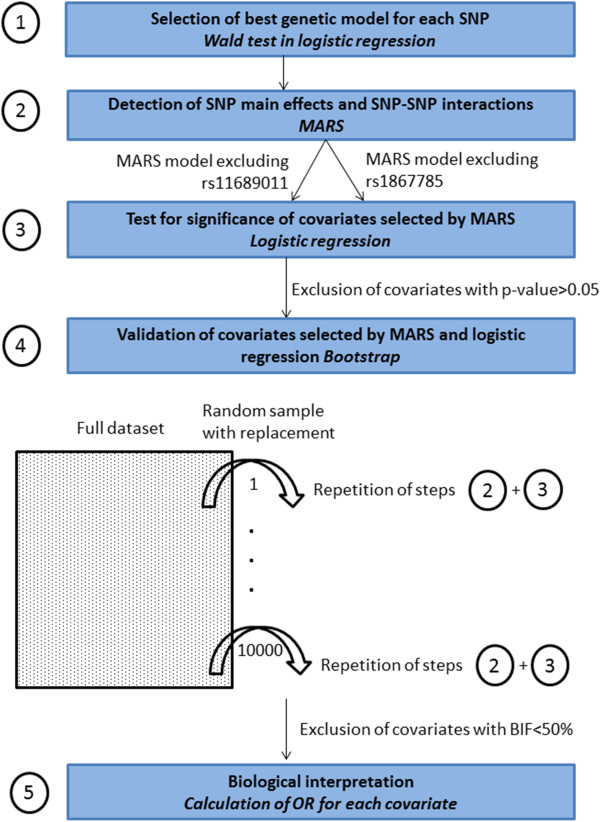
**The five steps that were followed in the statistical analysis.** MARS: Multivariate Adaptive Regression Splines, BIF: Bootstrap Inclusion Fraction. Firstly, we selected the best genetic model for each SNP by testing three inheritance models (dominant, recessive and additive model) for each SNP in the entire cohort of sprint/power athletes. Secondly, MARS was used to detect SNP main effects and SNP-SNP interactions (rs1867785 and rs11689011 were used in two independent MARS models because of their strong linkage disequilibrium). Thirdly, the covariates selected by MARS were input into a logistic regression model to determine their significance, and all covariates with p-value > 0.05 were excluded. Fourthly, to validate the selected covariates, we repeated steps 2 and 3 on 10000 random samples with replacement from the original dataset and calculated the how many times the selected covariates were significant in the 10000 random samples (BIF). All covariates with a BIF < 50% were excluded. Fifthly, we calculated the odds ratio of the genotype combinations for each selected covariate to give a clear biological interpretation.

The genotype frequencies of all individual SNPs are presented in Additional file 1. Before looking at both the individual effects of the five *EPAS1* SNPs and their interactions, we selected the best genetic model for each SNP. Then, SNP main effects, as well as SNP-SNP interactions, were investigated using Multivariate Adaptive Regression Splines (MARS), a nonparametric regression method [18] that has been successfully applied for detecting SNP-SNP interaction in several studies [19–22]. Finally, the odds ratios (OR) of being either a sprint/power athlete or an endurance athlete were calculated for each significant SNP and significant interacting pairs of SNPs using the best genetic model for each SNP. Details on the steps that have been taken in the statistical analysis are shown in Figure 1.

### Hardy–Weinberg equilibrium (HWE)

*χ*^2^ analysis was used to confirm if the control group, in each of the two samples, met HWE expectations. HWE was tested separately for each SNP.

### Selection of the best genetic model for each SNP

Three inheritance models (dominant, recessive and additive model) were assessed in the pooled cohorts of sprint/power and endurance athletes (Polish and Russian) using the Wald test in logistic regression models, and the best model was selected based on the minimum p-value for each SNP. The athletic status was divided into two categories and encoded as a dummy variable: athlete (encoded as 1) and control (encoded as 0). To adjust for multiple comparisons, the false discovery rate was controlled using the Benjamini and Hochberg correction [23]. For consistency, the same genetic model was applied separately in the Russian and Polish groups. All of the following steps were performed once when comparing sprint/power athletes with controls, and once again when comparing endurance athletes with controls.

### Analysis of SNP main effects and SNP-SNP interactions

Variable importance ranking for SNPs with strong Linkage Disequilibrium (LD) has been shown to be biased in previous studies [24, 25]. Therefore, LD in the 5 SNPs was examined using r^2^[26], and the pairs of SNPs displaying a strong LD (r^2^ > 0.8) were identified (Additional file 2). Only rs11689011 and rs1867785 were in strong LD (r^2^ = 0.96). Consequently, two MARS models, all corrected for sex, were developed: one including rs11689011, rs895436, rs4035887 and rs1867782, and another independent MARS model including rs1867785, rs895436, rs4035887 and rs1867782. For simplicity, we have only reported the MARS model excluding rs11689011 in the Results section.

In MARS, the maximum number of basis functions was set at 100, and the maximum degree of interaction was set at 2. As MARS does not provide variable significance using p-values, each covariate selected by MARS was used as input into a logistic regression model to determine its significance. All non-significant covariates (p-value > 0.05) were excluded. To confirm the significance of the covariates identified by the logistic regression, and to rank their importance, we used the Bootstrap Inclusion Fraction (BIF) criterion [27]. We obtained 10000 MARS models using 10000 random bootstrap samples with replacement from the original data set. Then, we calculated the proportion of times that a significant variable appeared in the 10000 MARS models and called this number the BIF. A variable which is approximately uncorrelated with others, and is only significant at the chosen nominal α level in the MARS model, is selected in about 50% of bootstrap samples. As the p-value diminishes, the BIF tends toward 100%. Thus, we only included covariates with a BIF > 50%.

For each significant covariate, the odds ratio (OR) of being either a sprint/power or an endurance athlete, depending on the genotype, was calculated. The 95% confidence intervals (CI) were calculated by unconditional maximum likelihood estimation.

## Results

In the pooled cohort of Russian and Polish controls, genotype distributions for each of the five SNPs was in agreement with HWE (p-value > 0.05).

### Endurance athletes vs. controls

In the Russian sample, the MARS model excluding rs11689011 yielded only one significant covariate, with a BIF > 50%. An interaction between sex and rs1867785 was observed: the GA and GG genotypes in rs1867785 were underrepresented in women endurance athletes (OR = 0.39, Table 2).

**Table 2:** **Covariates identified in the MARS model excluding rs11689011**

		Covariate	P-value^1^	BIF^2^	Odds Ratio	
Endurance athletes vs. controls	Russians	rs1867785*sex	0.00022	61.7	Other combinations	1 (ref)
					GA or GG in women	0.39 (0.24-0.65)
	Polish					
	Russians + Polish					
Sprint/power athletes vs. controls	Russians	rs4035887	0.0072	43.6	GA or GG	1 (ref)
					AA	0.54 (0.34-0.88)
		rs1867785	0.0017	78.3	GA or GG	1 (ref)
					AA	0.47 (0.25-0.84)
	Polish					
	Russians + Polish	rs1867785	0.00016	90.1	GA + GG	1 (ref)
					AA	0.53 (0.35-0.80)
		rs4035887*rs1867785	0.00016	52.6	Other combinations	1 (ref)
					AA at rs4035887 and	0.61 (0.45-0.85)
					GA or GG at rs1867785	

^1^P-value obtained by logistic regression.

^2^Bootstrap Inclusion Fraction calculated after running 10000 MARS models on 10000 bootstrap samples. A BIF of 90.1 indicates that the covariate of interest was selected in 90.1% of the MARS models.

*denotes an interaction.

In the Polish sample, no covariate was selected in the MARS model excluding rs11689011. When combining the Russian and the Polish groups, no covariate was selected in the MARS model excluding rs11689011.

Using the MARS model excluding rs1867785, the results were almost identical to those of the MARS model excluding rs11689011 (Additional file 3: Table S2). However, in the Polish sample one significant covariate was retained with a BIF > 50%. The TT genotype in rs11689011 was underrepresented in the cohort of endurance athletes (OR = 0.49, Additional file 3: Table S2), especially in the elite-level cohort compared to their sub-elite counterparts (OR = 0.31, 95% confidence interval: 0.11-0.87, Table 3).

**Table 3:** **Genotype frequencies of the three Single Nucleotide Polymorphisms (SNPs) significantly associated with athletic performance**

SNP	Major/minor allele	Model	Genotypes	Russians (Males + Females)					Polish (Males)				
				Controls (n = 175)	Endurance athletes (n = 148)		Sprint/power athletes (n = 246)		Controls (n = 428)	Endurance athletes (n = 106)		Sprint/power athletes (n = 92)	
rs11689011	T/C	Recessive	TC or CC	139 (79.4%)	119 (80.4%)		220 (89.4%)		353 (82.5%)	96 (90.6%)		82 (89.1%)	
					Elite	18 (72%)	Elite	97 (91.5%)		Elite	61 (93.8%)	Elite	53 (91.4%)
					Sub-elite	101 (82.1%)	Sub-elite	123 (87.9%)		Sub-elite	35 (85.4%)	Sub-elite	29 (85.3%)
			TT	36 (20.6%)	29 (19.6%)		26 (10.6%)		75 (17.5%)	10 (9.4%)		10 (10.9%)	
					Elite	7 (28%)	Elite	9 (8.5%)		Elite	4 (6.2%)	Elite	5 (8.6%)
					Sub-elite	22 (17.9%)	Sub-elite	17 (12.1%)		Sub-elite	6 (14.6%)	Sub-elite	5 (14.7%)
rs4035887	G/A	Dominant	GA or GG	130 (74.3%)	119 (80.4%)		207 (84.1%)		297 (69.4%)	68 (64.2%)		62 (67.4%)	
					Elite	19 (76.0%)	Elite	88 (83.0%)		Elite	41 (63.1%)	Elite	37 (63.8%)
					Sub-elite	100 (81.3%)	Sub-elite	119 (85.0%)		Sub-elite	27 (65.9%)	Sub-elite	25 (73.5%)
			AA	45 (25.7%)	29 (19.6%)		39 (15.9%)		131 (30.6%)	38 (34.9%)		30 (32.6%)	
					Elite	6 (24.0%)	Elite	18 (17.0%)		Elite	24 (36.9%)	Elite	21 (36.2%)
					Sub-elite	23 (18.7%)	Sub-elite	21 (15.0%)		Sub-elite	14 (31.4%)	Sub-elite	9 (26.5%)
rs1867785	A/G	Recessive	GA or GG	142 (81.1%)	122 (82.4%)		222 (90.2%)		356 (83.2%)	96 (90.6%)		82 (89.1%)	
					Elite	18 (72%)	Elite	98 (92.5%)		Elite	61 (93.8%)	Elite	53 (91.4%)
					Sub-elite	104 (84.6%)	Sub-elite	124 (88.6%)		Sub-elite	35 (85.4%)	Sub-elite	29 (85.3%)
			AA	33 (18.9%)	26 (17.6%)		24 (9.8%)		72 (16.8%)	10 (9.4%)		10 (10.9%)	
					Elite	7 (28%)	Elite	8 (7.5%)		Elite	4 (6.2%)	Elite	5 (8.6%)
					Sub-elite	19 (15.4%)	Sub-elite	16 (11.4%)		Sub-elite	6 (14.6%)	Sub-elite	5 (14.7%)

### Sprint/power athletes vs. controls

**Figure 2:**
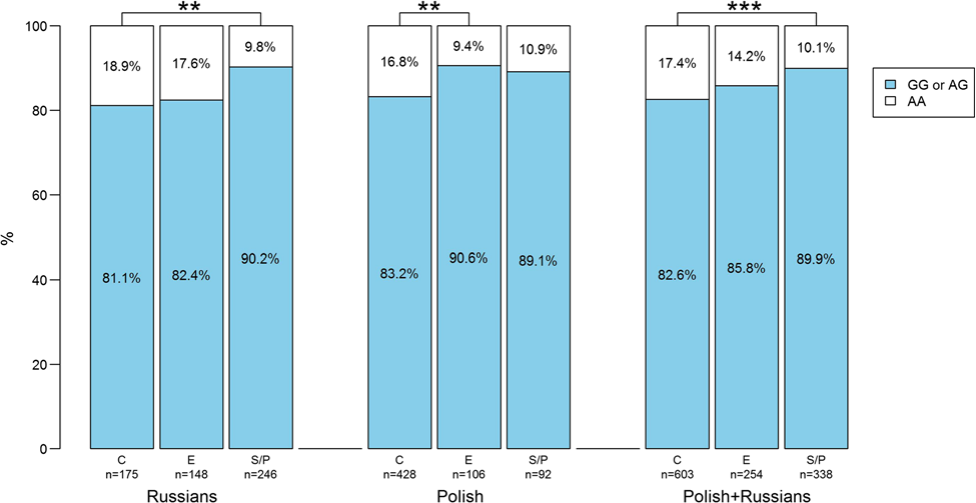
**Genotype distributions of rs1867785 in the different groups.** C: controls, E: endurance athletes, S/P: sprint/power athletes, **: p < 0.01 in linear regression, ***: p < 0.001 in linear regression.

In the Russian sample, the MARS model excluding rs11689011 yielded two significant covariates (p-value < 0.05 in logistic regression); only one covariate, however, had a BIF > 50% (Table 2). The AA genotype in rs1867785 was underrepresented in sprint/power athletes (OR = 0.47, Figure 2), especially in the elite sprint/power athletes compared to their sub-elite counterparts (OR = 0.35, 95% confidence interval: 0.16-1.79, Table 3). In the Polish sample, no covariates were selected in the MARS model (Table 2).

**Figure 3:**
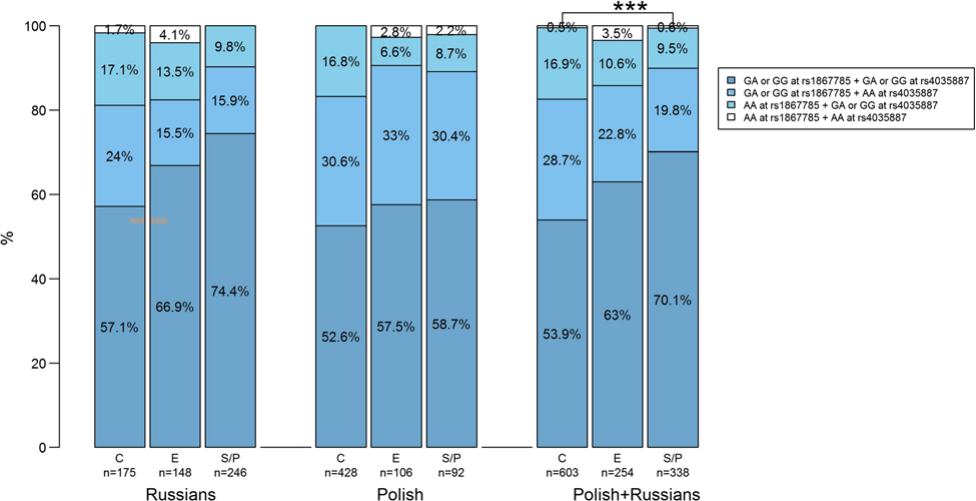
**Interaction between rs11689011, rs4035887 and athletic status in the different groups.** C: controls, E: endurance athletes, S/P: sprint/power, ***: p < 0.001 in linear regression.

In the Russian and Polish samples combined, the MARS model excluding rs11689011 yielded three significant covariates, two had a BIF > 50% (Table 2). The trend already observed for rs1867785 in the Russian sprint/power group was even stronger when combined with the Polish group (BIF = 90.1%); the AA genotype in rs1867785 was underrepresented in sprint/power athletes (OR = 0.53, Figure 2), especially in elite sprint/power athletes compared to their sub-elite counterparts (OR = 0.41, 95% confidence interval: 0.22-0.75). Furthermore, an interaction between rs1867785 and rs4035887 was found; the combination of the AA genotype in rs4035887 and the GA or GG genotype in rs1867785 was underrepresented in sprint/power athletes (OR = 0.61, Table 2 and Figure 3). We note that the genotype distribution of individual SNPs does not provide information regarding the genotype distribution of their combinations. For example, SNP_A_ and SNP_B_ are two SNPs with alleles A/a and B/b, respectively. In this example allele A is advantageous to performance, and allele B is also advantageous to performance but only when combined with allele a. From this pattern, one would expect allele A to be overrepresented in athletes, but the A + B combination to be underrepresented in athletes.

Using the MARS model excluding rs1867785, the results were almost identical to those of the MARS model excluding rs11689011 (Additional file 3: Table S2). However, the interaction between rs11689011 and rs4035887 in the Russian and Polish sample combined was not significant (BIF = 49.5%).

## Discussion

We have examined the genotype distribution and SNP-SNP interaction of five SNPs in the first intron of the *EPAS1* gene in European sprint/power and endurance athletes. We initially hypothesised that these SNPs would be associated with endurance athletic status. However, contrary to our hypotheses our main findings were as follow: In the pooled cohort of Polish and Russian athletes, 1) rs1867785 was associated with sprint/power athletic status; the AA genotype in rs1867785 was underrepresented in sprint/power athletes, 2) rs11689011 was also associated with sprint/power athletic status; the TT genotype in rs11689011 was underrepresented in sprint/power athletes, and 3) the interaction between rs1867785/rs11689011, and rs4035887 was associated with sprint/power athletic performance; the combinations of the AA genotype in rs4035887 with either the GA or GG genotypes in rs1867785, or with the CT or CC genotypes in rs11689011, were underrepresented in two cohorts of sprint/power athletes.

Compared with a previous study, the results of the present study were unexpected. Henderson et al. [12] have reported that the G allele in rs1867785 and the T allele in rs11689011 were overrepresented in endurance athletes, whereas we have observed that the G allele in rs1867785 was underrepresented (and the A allele was overrepresented) in female Russian endurance athletes. Furthermore, in our cohort, the T allele in rs11689011 was overrepresented in female Russian endurance athletes, but underrepresented in male Polish endurance athletes. A possible explanation for the discrepancies between our study and the study by Henderson et al. [12] is that our results regarding rs11689011 and rs1867785 were sex-specific. Sex-specific effects of SNPs are common in a wide range of phenotypes such as waist-hip ratio [28], susceptibility to sport-related injury [29], and increased muscle strength in response to training [30]. We have also recently shown that *PPARGC1A* rs4697425 was associated with elite female, but not with male endurance running performance [31]. However, the Polish endurance sample only comprised males, while the Russian sample included both males and females. Interestingly, the cohort in the study by Henderson et al. included both males and females athletes, but sex was not investigated as a covariate in their analysis [12].

Looking at each SNP separately, we found that rs1867785 and rs11689011 were associated with sprint/power performance. However, these SNPs had very similar genotype distribution and are in strong LD (r^2^ = 0.964); the A allele in rs1867785 segregates with the T allele in rs11689011. Since previous studies have suggested that SNPs with strong LD cannot be considered in the same statistical model [24, 25], and only one of these SNPs might influence sprint/power performance, we have created two MARS models and considered only one of the SNPs in each model. As expected, the two MARS models yielded almost identical results in all cohorts. However, only rs11689011 was associated with endurance athletic status in the Polish sample, while this was not the case for rs1867785. This might be explained by the small difference in genotype distribution in rs11689011 and rs1867785 in the Polish control sample. In addition, the BIF obtained for rs11689011, when comparing Polish endurance athletes with controls, was not strong enough (65.4%), indicating a correlation close to non-significance and sensitive to small changes in genotype frequencies. The relatively large sample size generated from studying two cohorts of athletes in the present study further reinforces the confidence in the results of the present study.

Indeed, in the present study two European Caucasian cohorts of athletes were grouped to explore the association between SNPs in *EPAS1* and athletic performance. Our previous results indicated that combining two cohorts of Caucasian athletes, especially when they are closely-related, would be useful approach to detect an association between SNPs and athletic status [13]. While previous studies combined athletes from different ancestries [32, 33], here we have studied athletes from closely-related European ancestries (e.g., Polish and Russians). This is reinforced by the similarity in the genotype distribution in the control groups (no more than 5% differences between the Russian and Polish controls). Furthermore, studying two cohorts of athletes has increased the sample size (overall 338 sprint/power athletes and 254 endurance athletes), which further strengthened our results and the likelihood that these specific *EPAS1* SNPs show a genuine association with elite sprint/power performance.

An additional novel finding in the present study is that the AA genotype in rs1867785, and the TT genotype in rs11689011, is even more underrepresented in elite sprint/power athletes compared with their sub-elite counterparts. This has previously been demonstrated for the highly-studied *ACTN3* R577X SNP, as the 577XX genotype was found in a lower frequency in elite sprint/power athletes compared to their national-level counterparts [34–36]. This observation indicates that while the *EPAS1* SNPs are associated with the development of sprint/power ability, they might be even more important in the development of world-class sprint/power ability. This finding, along with all the other findings in the present study, was obtained using the Bootstrap Inclusion Fractions (BIF) statistical method, which as far as we are aware of, has never been used in sports genomics.

The BIF analysis is a useful technique for investigating variations among selected models in samples drawn at random with replacement. Such samples mimic datasets that are structurally similar to that under study and that could plausibly have arisen instead [37]. Initially designed to test the stability of multivariable models, this non-parametric method allowed us to test whether the *EPAS1* SNPs selected by MARS were sensitive to small changes in the data, and confirm that they were unlikely to be false positives. Also, this method allowed us to see the relative importance of the different variables; while rs1867785 and rs11689011 showed very strong main effects in sprint/power athletes, their interaction with rs4035887 was of smaller importance.

We have also shown that several SNPs within *EPAS1* are associated with endurance athletic status, in a sex-specific manner. The TT genotype in rs11689011 was underrepresented in the cohort of Polish endurance athletes. However, this association was demonstrated only when either the Polish or the Russian groups were analysed separately, and was abolished when the two cohorts were combined. We argue that we cannot be certain that these positive findings are not false positives, as they might be a limitation of the smaller sample size. On the other hand, our positive findings regarding the association between the *EPAS1* SNPs and sprint/power athletic status were found in the combined cohort of sprint/power athletes, and the BIFs that were calculated for rs11689011 and rs1867785 were extremely high (90.1% for rs1867785, and 93.6% for rs11689011). Furthermore, these associations were more pronounced when the athletes’ level of competition was considered.

This study is not without limitations. In case–control studies, the relative proportions of controls and cases impacts the sample size required to detect an association with a given power and significance level. In the present study, with similar genotype distributions in Russians and Polish, to detect an association with the same effect size, at the same power and significance level, we would require a larger sample size in the Russian population. We acknowledge that the difference in numbers of athletes and controls in our study might therefore contribute to our results and the lack of replication in both athlete groups. However, in any association study with elite athletes it is a challenge to increase the sample size due to the very low number of elite athletes available to study.

Finally, in previous reports *EPAS1* SNPs have demonstrated an association with performance-related blood parameters (e.g., alterations in erythropoietin, hemoglobin and hematocrit) [9–11], and elite endurance performance [12], in humans. However, is has also been shown that *EPAS1* deficient mice have greater oxidative stress and an impaired response to oxidative stress [38]. A reduction in hematocrit levels and a global decrease in peripheral blood counts have also been observed in *EPAS1*-null mice [39]. Although no specific SNPs were tested in the mice model, these studies illustrate the potential importance of the *EPAS1* gene in athletic-related phenotypes.

## Conclusion

We found an association between *EPAS1* rs1867785 and *EPAS1* rs11689011 and sprint/power athletic status, and an interaction between rs1867785/11689011 and rs4035887 and sprint/power athletic status in two cohorts of closely-related European athletes. Based on the statistical model used either rs1867785 or rs11689011 are related to sprint/power athletic status. The association between rs1867785 and sprint/power athletic status is in line with a previous study in Australian athletes [12]. Unlike the vast number of investigations into the genetics of endurance performance, the genetic influence on elite sprint/power performance has received limited attention, and only a few studies have characterized the associations between genetic variants and elite sprint/power performance. Most studies to date have recruited only one cohort of athletes and were therefore hampered by insufficient sample size. In the present study, we have combined two cohorts of athletes and used innovative statistical methods, which provide confidence in our results. Functional studies directly demonstrating cause and effect, or providing any proposed cellular or molecular mechanisms to explain the association, are needed to extend and validate these findings.

## Electronic supplementary material

Additional file 1: **Genotype frequencies of the five investigated Single Nucleotide Polymorphisms (SNPs).** (DOCX 36 KB)

Additional file 2: **Linkage disequilibrium (LD) map of the five investigated SNPs in** ***EPAS1.*** The upper horizontal line represents the strand of chromosome 2 containing *EPAS1* and all five investigated SNPs. The triangle below indicates the pairwise LD (r^2^) between the five SNPs. Each SNP corresponds to a diagonal of this triangle, and the intersection of two diagonals contains the value of LD for the corresponding SNP pair. The colour within the squares represents the strength of the linkage between each pair. Of all possible pairs of SNPs, only rs1867785 and rs11689011 are in strong LD (red colour). (TIFF 17 MB)

Additional file 3: Table S2: Covariates identified in the different MARS models. (DOCX 20 KB)
