# Supplementary material for: EPAS1 gene variants are associated with sprint/power athletic performance in two cohorts of European athletes
Source: BMC Genomics. 2014 May 18;15(1):382. doi: 10.1186/1471-2164-15-382 (PMC4035083; doi:10.1186/1471-2164-15-382)
Supplement: Supplementary file 1 — Additional file 1: Genotype frequencies of the five investigated Single Nucleotide Polymorphisms (SNPs). (DOCX 36 KB) [file 12864_2013_6067_MOESM1_ESM.docx]

**Additional file 1. Genotype frequencies of the five investigated Single Nucleotide Polymorphisms (SNPs)**

| SNP | Major/minor allele | Genotypes | Russians | | | | | Polish | | | | |
| --- | --- | --- | --- | --- | --- | --- | --- | --- | --- | --- | --- | --- |
|  |  |  | Controls (n=175) | Endurance athletes (n=148) | | Sprint/power athletes (n=246) | | Controls (n=428) | Endurance athletes (n=106) | | Sprint/power athletes (n=92) | |
| rs895436 | C/G | CC | 45 (25.7%) | 39 (26.3%) | | 66 (26.8%) | | 88 (20.6%) | 18 (17.0%) | | 20 (21.7%) | |
|  |  |  |  | *elite* | 9 (36.0%) | *elite* | 29 (27.4%) |  | *elite* | 10 (15.4%) | *elite* | 10 (17.2%) |
|  |  |  |  | *sub-elite* | 30 (24.4%) | *sub-elite* | 37 (26.4%) |  | *sub-elite* | 8 (19.5%) | *sub-elite* | 10 (29.4%) |
|  |  | CG | 88 (50.3%) | 75 (50.7%) | | 128 (52.0%) | | 221 (51.6%) | 64 (60.4%) | | 43 (46.7%) | |
|  |  |  |  | *elite* | 12 (48.0%) | *elite* | 56 (52.8%) |  | *elite* | 42 (64.6%) | *elite* | 29 (50.0%) |
|  |  |  |  | *sub-elite* | 63 (51.2%) | *sub-elite* | 72 (51.4%) |  | *sub-elite* | 22 (53.7%) | *sub-elite* | 14 (41.2%) |
|  |  | GG | 42 (24.0%) | 34 (22.0%) | | 52 (21.2%) | | 119 (27.8%) | 24 (22.6%) | | 29 (31.5%) | |
|  |  |  |  | *elite* | 4 (16.0%) | *elite* | 21 (19.8%) |  | *elite* | 13 (20.0%) | *elite* | 19 (32.8%) |
|  |  |  |  | *sub-elite* | 30 (24.4%) | *sub-elite* | 31 (22.1%) |  | *sub-elite* | 11 (26.8%) | *sub-elite* | 10 (29.4%) |
| rs1867782 | C/G | CC | 157 (89.7%) | 133 (89.9%) | | 221 (89.8%) | | 379 (88.6%) | 93 (87.8%) | | 81 (88.0%) | |
|  |  |  |  | *elite* | 23 (92.0%) | *elite* | 98 (92.5%) |  | *elite* | 58 (89.2%) | *elite* | 51 (87.9%) |
|  |  |  |  | *sub-elite* | 110 (89.4%) | *sub-elite* | 123 (87.9%) |  | *sub-elite* | 35 (85.4%) | *sub-elite* | 30 (88.2%) |
|  |  | CG | 17 (9.7%) | 15 (10.1%) | | 23 (9.4%) | | 49 (11.4%) | 12 (11.3%) | | 11 (12.0%) | |
|  |  |  |  | *elite* | 2 (8.0%) | *elite* | 8 (7.5%) |  | *elite* | 6 (9.2%) | *elite* | 7 (12.1%) |
|  |  |  |  | *sub-elite* | 13 (10.6%) | *sub-elite* | 15 (10.7%) |  | *sub-elite* | 6 (14.6%) | *sub-elite* | 4 (11.8%) |
|  |  | GG | 1 (0.6%) | 0 (0%) | | 2 (0.8%) | | 0 (16.8%) | 1 (0.9%) | | 0 (10.9%) | |
|  |  |  |  | *elite* | 0 (0%) | *elite* | 0 (0%) |  | *elite* | 1 (1.5%) | *elite* | 0 (0%) |
|  |  |  |  | *sub-elite* | 0 (0%) | *sub-elite* | 2 (1.4%) |  | *sub-elite* | 0 (0%) | *sub-elite* | 0 (0%) |
| rs11689011 | T/C | TT | 36 (20.6%) | 29 (19.6%) | | 26 (10.6%) | | 75 (17.5%) | 10 (9.4%) | | 10 (10.9%) | |
|  |  |  |  | *elite* | 7 (28%) | *elite* | 9 (8.5%) |  | *elite* | 4 (6.2%) | *elite* | 5 (8.6%) |
|  |  |  |  | *sub-elite* | 22 (17.9%) | *sub-elite* | 17 (12.1%) |  | *sub-elite* | 6 (14.6%) | *sub-elite* | 5 (14.7%) |
|  |  | TC | 74 (42.3%) | 72 (48.6%) | | 125 (50.8%) | | 199 (46.5%) | 48 (45.3%) | | 45 (48.9%) | |
|  |  |  |  | *elite* | 8 (32.0%) | *elite* | 55 (51.9%) |  | *elite* | 31 (47.7%) | *elite* | 30 (51.7%) |
|  |  |  |  | *sub-elite* | 64 (52.0%) | *sub-elite* | 70 (50.0%) |  | *sub-elite* | 17 (41.5%) | *sub-elite* | 15 (44.1%) |
|  |  | CC | 65 (37.1%) | 47 (31.8%) | | 95 (38.6%) | | 154 (36.0%) | 48 (45.3%) | | 37 (40.2%) | |
|  |  |  |  | *elite* | 10 (40.0%) | *elite* | 42 (39.6%) |  | *elite* | 30 (46.2%) | *elite* | 23 (39.7%) |
|  |  |  |  | *sub-elite* | 37 (30.1%) | *sub-elite* | 53 (37.9%) |  | *sub-elite* | 18 (43.9%) | *sub-elite* | 14 (41.2%) |
| rs4035887 | G/A | GG | 45 (25.7%) | 30 (20.3%) | | 52 (21.1%) | | 78 (18.2%) | 13 (12.3%) | | 14 (15.2%) | |
|  |  |  |  | *elite* | 9 (36.0%) | *elite* | 21 (19.8%) |  | *elite* | 10 (15.4%) | *elite* | 10 (17.2%) |
|  |  |  |  | *sub-elite* | 21 (17.1%) | *sub-elite* | 31 (22.1%) |  | *sub-elite* | 3 (7.3%) | *sub-elite* | 4 (11.8%) |
|  |  | GA | 85 (48.6%) | 89 (60.1%) | | 155 (63.0%) | | 219 (51.2%) | 55 (51.9%) | | 48 (52.2%) | |
|  |  |  |  | *elite* | 10 (40.0%) | *elite* | 67 (63.2%) |  | *elite* | 31 (47.7%) | *elite* | 27 (46.6%) |
|  |  |  |  | *sub-elite* | 79 (64.2%) | *sub-elite* | 88 (62.9%) |  | *sub-elite* | 24 (58.5%) | *sub-elite* | 21 (61.7%) |
|  |  | AA | 45 (25.7%) | 29 (19.6%) | | 39 (15.9%) | | 131 (30.6%) | 38 (34.9%) | | 30 (32.6%) | |
|  |  |  |  | *elite* | 6 (24.0%) | *elite* | 18 (17.0%) |  | *elite* | 24 (36.9%) | *elite* | 21 (36.2%) |
|  |  |  |  | *sub-elite* | 23 (18.7%) | *sub-elite* | 21 (15.0%) |  | *sub-elite* | 14 (31.4%) | *sub-elite* | 9 (26.5%) |
| rs1867785 | A/G | AA | 33 (18.9%) | 26 (17.6%) | | 24 (9.8%) | | 72 (16.8%) | 10 (9.4%) | | 10 (10.9%) | |
|  |  |  |  | *elite* | 7 (28.0%) | *elite* | 8 (7.5%) |  | *elite* | 4 (6.2%) | *elite* | 5 (8.6%) |
|  |  |  |  | *sub-elite* | 19 (15.4%) | *sub-elite* | 16 (11.4%) |  | *sub-elite* | 6 (14.6%) | *sub-elite* | 5 (14.7%) |
|  |  | AG | 73 (41.7%) | 75 (50.7%) | | 122 (49.6%) | | 202 (47.2%) | 48 (45.3%) | | 46 (50.0%) | |
|  |  |  |  | *elite* | 8 (32.0%) | *elite* | 53 (50.0%) |  | *elite* | 31 (46.7%) | *elite* | 31 (53.4%) |
|  |  |  |  | *sub-elite* | 67 (54.5%) | *sub-elite* | 69 (49.3%) |  | *sub-elite* | 17 (41.5%) | *sub-elite* | 15 (44.1%) |
|  |  | GG | 69 (39.4%) | 47 (31.7%) | | 100 (40.7%) | | 154 (36.0%) | 48 (45.3%) | | 36 (39.1%) | |
|  |  |  |  | *elite* | 10 (40.0%) | *elite* | 45 (42.5%) |  | *elite* | 30 (41.2%) | *elite* | 22 (37.9%) |
|  |  |  |  | *sub-elite* | 37 (30.1%) | *sub-elite* | 55 (39.3%) |  | *sub-elite* | 18 (43.9%) | *sub-elite* | 14 (41.2%) |
