# Supplementary figures and images for: EPAS1 gene variants are associated with sprint/power athletic performance in two cohorts of European athletes
Source: BMC Genomics. 2014 May 18;15(1):382. doi: 10.1186/1471-2164-15-382 (PMC4035083; doi:10.1186/1471-2164-15-382)

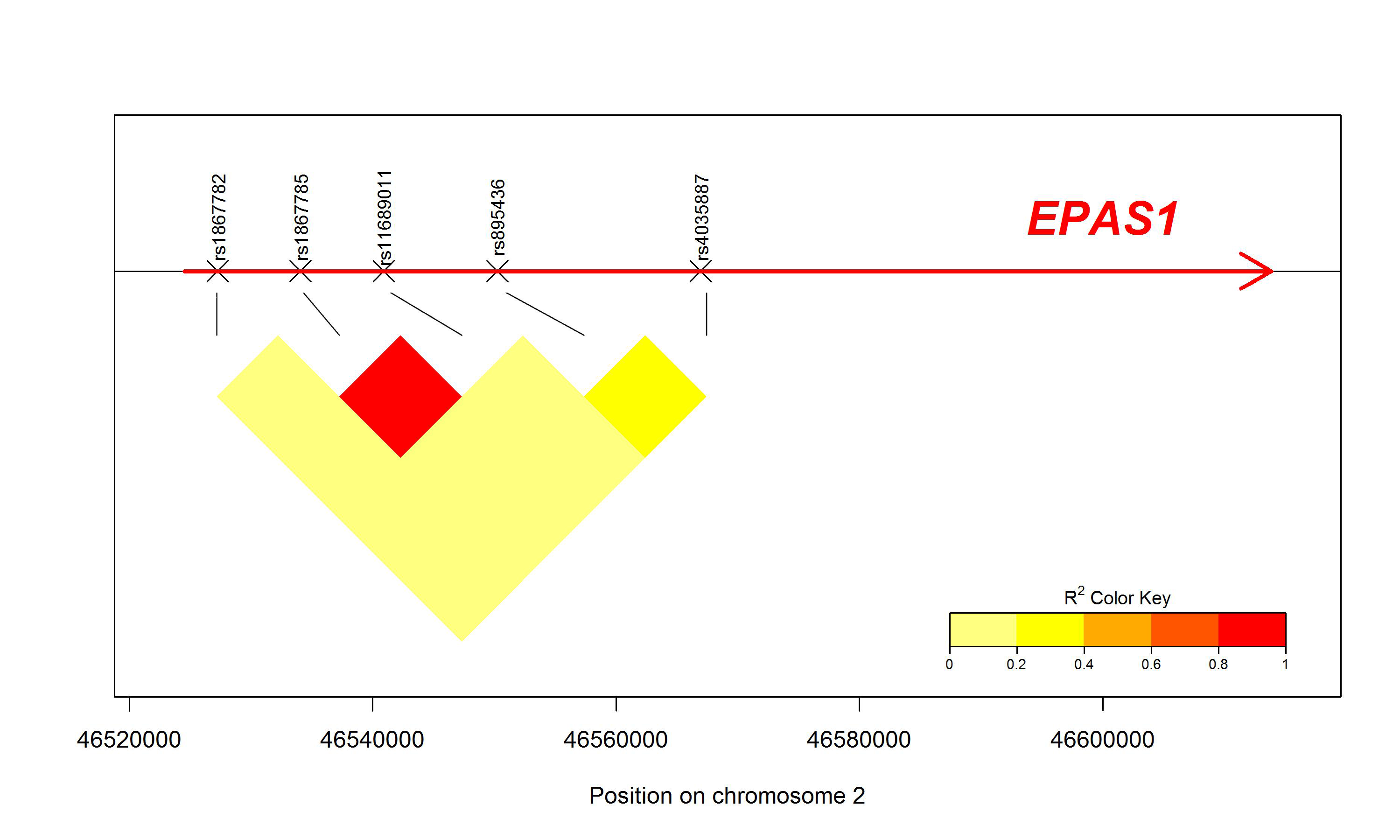

Supplement: Supplementary file 2 — Additional file 2: Linkage disequilibrium (LD) map of the five investigated SNPs in EPAS1. The upper horizontal line represents the strand of chromosome 2 containing EPAS1 and all five investigated SNPs. The triangle below indicates the pairwise LD (r2) between the five SNPs. Each SNP corresponds to a diagonal of this triangle, and the intersection of two diagonals contains the value of LD for the corresponding SNP pair. The colour within the squares represents the strength of the linkage between each pair. Of all possible pairs of SNPs, only rs1867785 and rs11689011 are in strong LD (red colour). (TIFF 17 MB) [file 12864_2013_6067_MOESM2_ESM.tiff]
