# Supplementary material for: EPAS1 gene variants are associated with sprint/power athletic performance in two cohorts of European athletes
Source: BMC Genomics. 2014 May 18;15(1):382. doi: 10.1186/1471-2164-15-382 (PMC4035083; doi:10.1186/1471-2164-15-382)
Supplement: Supplementary file 3 — Additional file 3: Table S2: Covariates identified in the different MARS models. (DOCX 20 KB) [file 12864_2013_6067_MOESM3_ESM.docx]

**Additional file 3: Table S2 Covariates identified in the different MARS models.**

|  | |  | MARS model excluding rs11689011 | | | | | MARS model excluding rs1867785 | | | | |
| --- | --- | --- | --- | --- | --- | --- | --- | --- | --- | --- | --- | --- |
|  | | | Covariate | P-value^1^ | BIF^2^ | OR | | Covariate | P-value^1^ | BIF^2^ | OR | |
| Endurance athletes vs. controls | Russians | | rs1867785*sex | 0.00022 | 61.7 | other combinations | 1 (ref) | rs11689011*sex | 0.00013 | 54.9 | other combinations | 1 (ref) |
|  |  |  |  |  |  | GA or GG in women | 0.39 (0.24-0.65) |  |  |  | CT or CC in women | 0.38 (0.23-0.62) |
|  | Polish | |  |  |  |  |  | rs11689011 | 0.0059 | 65.4 | CT or CC | 1 (ref) |
|  |  |  |  |  |  |  |  |  |  |  | TT | 0.49 (0.24-0.98) |
|  | Russians + Polish | |  |  |  |  |  |  |  |  |  |  |
|  |  | | | | | | | | | | | |
| Sprint/power athletes vs. controls | Russians | | rs4035887 | 0.0072 | 43.6 | GA or GG | 1 (ref) | rs4035887 | 0.0071 | 41.3 | GA or GG | 1 (ref) |
|  |  |  |  |  |  | AA | 0.54 (0.34-0.88) |  |  |  | AA | 0.54 (0.34-0.88) |
|  |  |  | rs1867785 | 0.0017 | 78.3 | GA or GG | 1 (ref) | rs11689011 | 0.0011 | 84.4 | CT or CC | 1 (ref) |
|  |  |  |  |  |  | AA | 0.47 (0.25-0.84) |  |  |  | TT | 0.46 (0.26-0.79) |
|  | Polish | |  |  |  |  |  |  |  |  |  |  |
|  | Russians + Polish | | rs1867785 | 0.00016 | 90.1 | GA+GG | 1 (ref) | rs11689011 | 0.00011 | 93.6 | CT or CC | 1 (ref) |
|  |  |  |  |  |  | AA | 0.53 (0.35-0.80) |  |  |  | TT | 0.52 (0.35-0.79) |
|  |  |  | rs4035887*rs1867785 | 0.00016 | 52.6 | other combinations | 1 (ref) | rs4035887*rs11689011 | 0.00016 | 49.5 | other combinations | 1 (ref) |
|  |  |  |  |  |  | AA at rs4035887 and | 0.61 (0.45-0.85) |  |  |  | AA at rs4035887 and | 0.62 (0.45-0.85) |
|  |  |  |  |  |  | GA or GG at rs1867785 |  |  |  |  | CT or CC at rs11689011 |  |

^1^P-value obtained by logistic regression

^2^Bootstrap Inclusion Fraction calculated after running 10000 MARS models on 10000 bootstrap samples. A BIF of 90.1 indicates that the covariate of interest was selected in 90.1% of the MARS models.
